# Water deprivation in poultry in connection with transport to slaughter—a review

**DOI:** 10.1016/j.psj.2023.103419

**Published:** 2023-12-30

**Authors:** K.E. Wurtz, M.S. Herskin, A.B. Riber

**Affiliations:** Department of Animal and Veterinary Sciences, Aarhus University, DK-8830 Tjele, Denmark

**Keywords:** broiler, laying hen, welfare, physiology, production

## Abstract

Poultry are deprived of water when transported to slaughter, beginning shortly prior to catching of the first bird and lasting through catching and loading, the journey on the vehicle, time spent in lairage, and up until time of death. Our aim was to review existing knowledge on variables which may be useful in determining the length of time that poultry may go without water in connection with transport before their welfare begins to deteriorate. During transport, it is likely that birds experience a motivation to drink, which may transition into the negative emotional state of thirst if water is unavailable. Determining when drinking motivation reaches a threshold where welfare is negatively impacted is challenging. In the absence of water, birds may over time experience dehydration which may be detected through physiological indicators as their body attempts to maintain homeostasis. In poultry, plasma osmolality, arginine vasotocin, and chloride have been suggested as being most suitable for assessing dehydration resulting from periods of water deprivation that correspond with typical transport durations, due to their particular sensitivity during this period. While initial dehydration may not be associated with negative emotional states, it is likely that it eventually leads to discomfort, but additional behavioral and motivational studies are necessary to infer when this begins. Impacts of thermal conditions, genetics, and the condition of the individual bird on the development of a dehydrated state were also assessed, though more information is needed to fully understand these interactions. With the available literature, this review concludes that total transport (i.e., from the initial deprivation from water until time of slaughter) durations of longer than 6 h are likely associated with measurable physiological indicators of dehydration and may potentially be associated with negative emotional states, although more research is needed to clarify this. Current available knowledge and assessment tools are not sufficient to detect the degradation of welfare derived from thirst itself, which should be further examined to protect poultry welfare during transport.

## INTRODUCTION

In poultry production, transport to slaughter represents a critical period in regard to risk of experiencing dehydration or thirst due to the standard practice of preslaughter water deprivation (Vanderhasselt et al., 2013). Water and feed are typically withheld from poultry prior to and during transport to reduce the risk of fecal contamination of the carcasses during processing and to help keep the birds clean and dry during transport (European Commission, 2004; Warriss et al., 2004; Rasschaert et al., 2020). Legislation and animal welfare assurance schemes may limit the distances or duration that livestock and poultry may be transported in an effort to safeguard animal welfare. However, the total time that birds are deprived of water in connection with transport substantially exceeds the time in transit (i.e., the time on the vehicle excluding loading and unloading time; deemed “journey time” throughout this review) (EFSA AHAW Panel, 2022), and thus limiting journey time in an effort to minimize the experience of negative emotional states associated with transport is not enough. Instead, the entire period associated with transport to slaughter, including preparation, catching, loading, journey, and lairage, must be fully considered. In addition, factors such as environmental conditions, genetics, and individual bird condition must be taken into account when determining acceptable transport durations.

Thirst has been defined as a negative motivational state which may arise to drive water seeking behavior (Mellor, 2017) if access to water is limited or restricted (Jensen and Vestergaard, 2021). It has been theorized that thirst intensity increases as the threat to fitness (e.g., risk of severe dehydration) increases (Fraser and Duncan, 1998). Without the ability to drink, birds are at risk of experiencing negative emotions as well as a net loss of total body water putting them in a dehydrated state (Thomas et al., 2008). While the definition of emotion is inconsistent throughout the literature (Kremer et al., 2020), we have chosen to align our writing with the proposed definition by Paul and Mendl (2018) which states that for animals, an emotion is a multicomponent (subjective, physiological, behavioral, and cognitive) response to a stimulus or event that is of importance to the individual which is always valenced and can vary in activation/arousal and duration/persistence. The progression of the experience of thirst relative to the extent of dehydration remains unclear, and likely depends on a combination of multiple sensory and feedback mechanisms. It is, for example, understood that neurons located within the lamina terminalis in the brain sense changes in plasma osmolality and respond by releasing hormones that either stimulate or inhibit drinking motivation (McKinley and Johnson, 2004; Augustine et al., 2018). It is, however, not that simple, as animals appear to retain a behavioral motivation to drink even in the absence of a physiological need for water. For example, rats provided with sufficient water intravenously to meet their physiological needs still voluntarily consume water orally (Nicolaidis and Rowland, 1975). Studies conducted on other species show that drinking behavior in itself is rewarding as it released dopamine (Augustine et al., 2019). Therefore, there appears to be both physiological and behavioral mechanisms that motivate drinking behavior. The motivation to drink may be assessed behaviorally, using motivational tests or measures of voluntary water intake. Dehydration, which may also be associated with negative emotional states, may be detected by physiological indicators, however, as mentioned the relationship between the degree of dehydration and the degree of thirst is not clear.

According to the FAO, approximately 70 billion broiler chickens are produced globally per year (FAO, 2023) and virtually all of these individuals will be transported to slaughter plants once they reach a marketable weight. Other poultry species, such as end-of-lay hens, ducks, geese, turkeys, quail, and game birds may similarly be subjected to transport at various points in their lives. Though transport makes up a small percentage of an individual's lifespan, the potential for severe negative welfare consequences during this period is high. For instance, some studies have found that longer journey durations are associated with increased dead on arrival (**DOA**) rates (e.g., Kittelsen et al., 2015; Vecerek et al., 2016; Saraiva et al., 2020), and the conditions leading up to death are likely to be perceived as negative by the individuals.

Increased mortality, combined with reductions in carcass quality resulting from dehydration have serious economic implications that should be considered (Speer et al., 2001). In broilers, global DOA percentages can vary substantially but averages ranging from 0.11 to 0.68% have been reported in studies (Vieira et al., 2011; Jacobs et al., 2017a). Based on the annual global production of broilers, this would correspond to roughly 77 to 476 million broilers per year. While other factors, such as heat stress or physical injuries resulting from rough handling, have been shown to have a greater influence on mortality, dehydration resulting from water deprivation may exacerbate underlying health conditions, therefore indirectly contributing to increased mortality. Thus, even though water deprivation is not the major cause of DOA, the potential combined effects of this on animal welfare, economics, and sustainability are considerable.

Determining the ideal recommended maximum time that poultry can go without water is challenging. Cockram (2007) suggested that the onset of physiological changes indicative of the initiation of homeostatic mechanisms to preserve water could be used as an early indicator of whether a period of water deprivation has an effect. However, these indicators are not necessarily associated with emotions which, depending on the framework used to assess animal welfare, may limit their usefulness for making decisions regarding maximum transport durations to avoid negative impacts to welfare. Further assessment is required to determine the point at which welfare begins to deteriorate in relation to these physiological variables. Evidence regarding the impact of water deprivation of various lengths on physiological measures, behavioral responses, and motivational measures are reviewed in this paper in an attempt to elucidate their interconnection. We suggest that durations of water deprivation that cause significant deviations in physiological measures from baseline values and that additionally show evidence of generating negative emotional states may be used in combination to help formulate recommended maximum transport durations.

The specific aims of this paper were to 1) describe the different phases of transport that poultry go through in connection to slaughter, with a focus on access to water, and 2) review existing knowledge on behavioral, physiological, and performance indicators of thirst and dehydration which may be useful in determining the length of time that poultry may go without water in connection with transport before their welfare becomes negatively affected. The focus of this review is on broiler chickens due to the availability of literature, however, relevant literature on laying hens, ducks, turkeys, and quail has also been included when available.

## MATERIALS AND METHODS

An initial search in June 2022 using Web of Science returned 15 articles using search terms of either “broiler,” “chicken,” “poultry,” “laying hen,” “duck,” or “turkey” with “thirst,” “water deprivation,” “deprivation of water,” “water withdrawal,” “welfare,” or “dehydration” with “slaughter and transport” or just “transport.” Checking references of these initial papers yielded an additional 10 relevant articles. Google Scholar was then searched using similar search terms which yielded 31 additional articles. Checking the references of these articles yielded an additional 4 articles. Additional articles were accumulated throughout the data extraction process through following citations of cited papers (snowballing). The EFSA AHAW Panel (2022) scientific opinion concerning the welfare of domestic birds and rabbits transported in containers was published after the initial literature search but has been included in the review as a reference as well.

## RESULTS

### Phases of Transport

The transport process which begins on farm and ends at slaughter may be classified into 4 distinct phases: preparation, catching and loading, journey, and lairage and unloading. Each phase presents its own unique welfare challenges to poultry (EFSA, 2011; Grandin, 2019; Jacobs and Tuyttens, 2020), but all share one common theme; the birds experience water deprivation.

***Preparation.*** To reduce the risk of fecal contamination of the carcasses during processing and to help maintain cleanliness of the birds during transport, feed and water are withheld from poultry prior to their journey to slaughter (European Commission, 2004; Warriss et al., 2004; Rasschaert et al., 2020). To ensure that most material has passed through the gastrointestinal tract before the journey is initiated, feed is typically removed from broilers for anywhere from 4 to 8 h prior to catching, and for 2 to 8 h prior to catching for turkeys (Bilgili, 2002; EFSA AHAW Panel, 2022). Meat ducks are typically fasted for at least 8 h, while quail may have feed withdrawn for between 6 to 8 h (EFSA AHAW Panel, 2022). While these estimates give a general guideline for typical fasting durations, variation exists in practice, and total feed withdrawal durations may be much longer (e.g., over 24 h for broilers in a Belgian study (Jacobs et al., 2017b)). Water will often continue to be offered right up until the first bird is caught to help clear any residual feed from the crop and proventriculus (Savage, 1995; Saraiva et al., 2020).

***Catching and Loading.*** Within the European Union, most poultry species are transported within containers (e.g., crates or modules). The birds may be either manually or mechanically caught on farm and placed into containers which are then loaded into vehicles approved for transport of poultry (Nijdam et al., 2004). Catching duration can vary considerably depending on the size of the flock, the housing system, and the method of catching utilized. It is estimated that 1,000 to 1,300 broilers may be caught per h per manual catcher (Metheringham and Hubrecht, 1996). In a study of commercial transport in Belgium (52 farms), average catching and loading time was found to be 4 h and 47 min (range: 1 h and 25 min to 11 h) with an average of 8 catchers (range: 3–12) (Jacobs et al., 2017b). Mechanical catching systems can catch birds at a rate of 5,000 to 6,000 per h (Lund et al., 2013).

***Journey.*** Before departure, the contained birds are loaded onto trucks and driven to the slaughter plant. The design of the transport system impedes provision of water on the vehicle (Rault et al., 2016). Due to EU transport regulations (EC 1/2005), most journeys within the EU are therefore limited to a maximum of 12 h, as water must be provided for journey times exceeding 12 h, exclusive of preparation, catching, loading, delays, and lairage time. In the EU, end-of-lay hens that are sent for slaughter often have longer journey times than broilers due to lack of available slaughter facilities dedicated to processing these hens (though journey time is still usually less than 12 h) (Weeks et al., 2012). On the other hand, broilers and turkeys generally have shorter journey times to slaughter due to the large availability of slaughter plants required to keep up with their large production volumes. Similarly, ducks typically have short journey times, with reported durations ranging from 0 to 3 h (EFSA AHAW Panel, 2022). However, the size of the country and availability of slaughter plants impacts journey time, meaning these numbers are only estimates. In addition, for all animal transport, journey times may be affected by traffic congestion, accidents, or other related hazards (Warriss et al., 1990).

***Lairage and Unloading.*** Upon arrival at the slaughter plant, poultry remain in the containers either on the truck or are unloaded and held until the slaughter line is ready to process them. Sometimes in hot environments, water may be misted or sprayed over the containers during lairage to aid in thermoregulation, which may allow for some water consumption by some of the birds (Komiyama et al., 2008). However, in the vast majority of cases, complete water deprivation continues through the lairage period. Generally, longer durations spent in lairage lead to increased percentages of DOA observed at slaughter (Nijdam et al., 2004; Rojas et al., 2010). In a study examining preslaughter factors affecting welfare of broilers in Portugal, an average lairage time of 6 h and 52 min (maximum time of 14 h and 39 min) was reported (Saraiva et al., 2020). In the UK, while the average journey time for broilers was 2 h and 42 min, when loading and lairage times were factored in, broilers spent on average 3 h and 36 min in transport, with 6% of loads taking over 7 h (Warriss et al., 1990). Birds transported to slaughter plants with smaller throughput were found to have longer durations on average of time spent contained (Warriss et al., 1990). Data from the transport of broilers in Belgium showed an average lairage time of 4 h and 35 min (Jacobs et al., 2017b).

Summing up, this means that even with relatively short journeys, birds may be without water for significant periods of time when durations of all the phases involved in transport are considered (EFSA AHAW Panel, 2022). In a study on preslaughter handling and transport of commercial broilers in Denmark, it was determined that the average time birds spent in containers was 5 h and 27 min (CI: 4 h and 20 min–6 h and 34 min) (Lund et al., 2013). In Belgium, an overall water withdrawal time was found to be 7 h and 20 min on average (Jacobs et al., 2017b). For reference, broilers on farm typically have constant access to water and have been shown to consume water on average 7.2 times per 4-h period (Christensen, 2010).

### Assessment of Dehydration and Thirst

For decades, freedom from thirst has been recognized as a condition imperative to good animal welfare (FAWC, 2009). To this day, organizations continue to prioritize freedom from prolonged thirst in their welfare assessment protocols and in reports on welfare consequences of transport, though what qualifies as “prolonged” is often not clearly defined (Tuyttens et al., 2010; EFSA AHAW Panel, 2022).

Signs of dehydration and/or thirst in poultry may be assessed by animal-based-measures (**ABMs**), of which some are behavioral, physiological, or performance related. So far, however, the vast majority of studies of these states have been carried out under production conditions or in experimental set-ups not related to transport and slaughter. Additionally, many of the studies assessing the effects of water deprivation provided feed to the birds, which would not be truly representative of conditions experienced during transport in which birds are deprived of both feed and water. Below, relevant results providing information on the length of time that poultry may go without water before their welfare is challenged is reviewed based on these 3 categories of ABMs.

***Behavioral Indicators.*** Behavioral measures provide a tool for assessing motivation as well as drawing inferences about emotional states (e.g., as reviewed by Weary et al., 2017). As an indicator of drinking motivation, compensatory drinking has been shown to increase proportionally with the duration of water deprivation from 6 to 24 h in broiler chickens (Sprenger et al., 2009; Vanderhasselt et al., 2014). In an operant conditioning test, Haskell et al. (2000,^2004^) found that water deprivation of periods of 2 and 6 h caused laying hens to display redirected aggression toward a subordinate individual when access to water in a dish was thwarted using a clear lid. Other behavioral changes, such as increased drinking duration (after 24–32 h of water deprivation), increased proximity to the drinkers (after 18 h of deprivation), and reduced standing (after 18 h of deprivation) have been observed in connection to water deprivation when periods of 0, 12, 18, 24, and 32 h were assessed (Rault et al., 2016). Interestingly, after 32 h of water deprivation, hens were observed spending considerable time pecking at the empty feeders while in a test arena, despite not experiencing any restriction to their feed while in their home pens (Rault et al., 2016). Birds voluntarily reduce their feed consumption in the absence of water (Ross et al., 1981), and thus the increased pecking at the feeder could have been a result of birds searching for feed once water was made available again. Alternatively, the increased pecking at the feeder could indicate a motivation to drink, as drinking and eating are highly linked behaviors that often occur in close connection (Savory et al., 1978).

Motivation tests can provide information regarding the strength of a bird's motivation to access water to drink (Kirkden and Pajor, 2006), and tests have demonstrated laying hens’ willingness to squeeze through narrow gaps to gain access to water after 12 h of water deprivation (birds were tested after 0, 12, 18, 24, and 32 h of deprivation) (Rault et al., 2016).

Tests have been developed to measure emotional states and preferences in poultry such as cognitive bias tests and conditioned place avoidance or preference tests (Jacobs et al., 2023), but, to the best of our knowledge, tests have yet to be applied to assess thirst. Given that thirst is one of the longest recognized welfare concerns, this absence in the literature may seem surprising.

***Physiological Indicators.*** Poultry have the ability to maintain plasma volume during periods of water deprivation to some extent. They do so in a similar manner as certain desert mammals through preferential loss of water from extravascular compartments, preserving vascular volume (Horowitz et al., 1978). In response to dehydration, plasma osmolarity increases, triggering the release of the antidiuretic hormone, arginine vasotocin (**AVT**) (Skadhauge, 2012). AVT reduces glomerular filtration rate and increases tubular water resorption, aiding the ability to concentrate urine (Skadhauge, 2012). Birds may further combat the effects of dehydration through water absorption in the lower intestine, which is thought to be regulated by prolactin, aldosterone, and corticosterone (Skadhauge, 2012). Effects of water deprivation of varying lengths on various physiological variables have been described in the literature for poultry species and are discussed in greater detail in the following sections. Unless otherwise noted, the studies reviewed below were conducted using poultry maintained within their thermoneutral zone.

**Figure 1:**
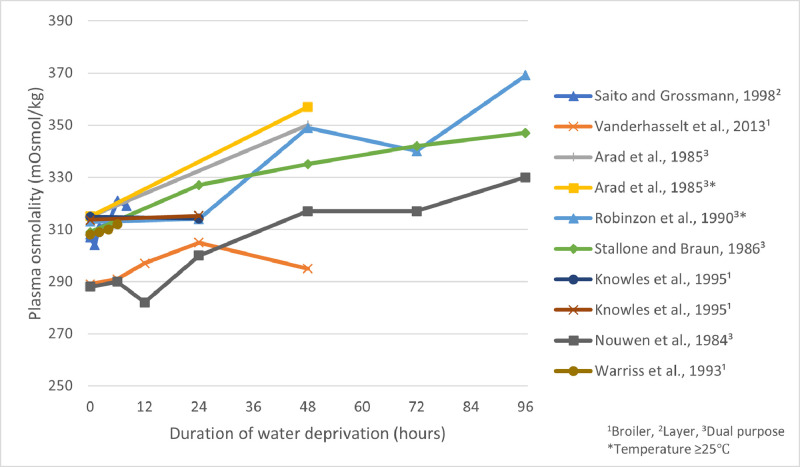
Plasma osmolality concentrations (mOsmol/kg) reported by various studies across various durations of water deprivation. Points correspond with the time in which the measure was taken and are connected by a linear line for illustrative purposes, though in reality these relationships are not necessarily linear in nature. The shortest duration of deprivation measured was 0.5 h (Saito and Grossmann, 1998).

Plasma osmolality measures the concentration of particles within the blood, and high osmolality (due to a reduction in plasma water content) can be indicative of dehydration and may be used to scientifically assess the effects of water deprivation that occur during the “catching to slaughter”-interval in poultry (Vanderhasselt et al., 2013). In a survey following the transport of 800 broilers to slaughter plants in the UK with journey times ranging from 0 h to 2 h and 36 min, broilers with longer journey times were found to have higher osmolality values (Knowles et al., 1996). Results from the literature regarding the duration of water deprivation that produce significant changes from baseline in plasma osmolality obtained under nontransport, resting conditions, are variable though and are likely influenced by factors such as temperature or genetics. For instance, Saito and Grossmann (1998) found that osmolality values were significantly higher in White Leghorn laying hens subjected to 8 h of water deprivation compared to hens in a control group not subjected to deprivation, however samples were not collected at the same time of day between treatment groups, potentially impacting the reliability of the results. Additionally, these birds retained access to feed, which may in part have explained the observed rise in plasma osmolality values (Swayne and Radin, 1991). Nouwen et al. (1984) did not detect significant increases in osmolality until after 24 h in a study with male and female Rhode Island Red chickens (layer strain) (Figure 1). It was hypothesized that this difference may have been due to the increased water requirements for egg production in the population of relatively high-yielding hens (vs. a mix of lower-yielding hens and roosters) (Saito and Grossmann, 1998). Vanderhasselt et al. (2013) observed steady increases in plasma osmolality in broilers over a 24 h period of water deprivation, which later decreased between h 24 and 48 of water withdrawal (Figure 1). The authors suggested this observed decrease could have been related to a reduction in feed intake associated with prolonged water deprivation (Koike et al., 1983), which in turn can decrease plasma osmolality values (Knowles et al., 1995). Other studies have found gradual increased plasma osmolality over longer time periods, such as over 48 h in White Plymouth Rock chickens (dual-purpose strain; with concentrations measured at 48 h being significantly higher than baseline) (Arad et al., 1985) and over 96 h of deprivation in White Leghorn cockerels (housed between 25°C and 27°C; though concentrations did not differ significantly from baseline when measured between 24 and 48 h) (Robinzon et al., 1990) and Rhode Island Red cockerels (significant differences from baseline observed after 24, 48, 72, and 96 h, though 24 h was the earliest time point assessed) (Stallone and Braun, 1986) (Figure 1). Warriss et al. (1993) did not observe significant changes in plasma osmolality during transport of broilers for 2, 4, or 6 h (Figure 1). Similarly, Knowles et al. (1995) did not observe significant changes in osmolality after 24 h of feed and water deprivation in broilers. Based on the literature, the shortest duration reported in which birds experienced a statistically significant change in their plasma osmolality from baseline was at 6 h (Saito and Grossmann, 1998), however, this study was conducted on laying hens, which may be more sensitive than broilers to water deprivation, and birds retained access to feed which may have contributed to a more rapid rise in plasma osmolality than what would be the case if feed was also withdrawn. The shortest duration noted in broilers in which there was a significant difference from values in control birds was 24 h (with measures taken at 6, 12, 24, and 48 h) (Nouwen et al., 1984; Vanderhasselt et al., 2013).

**Figure 2:**
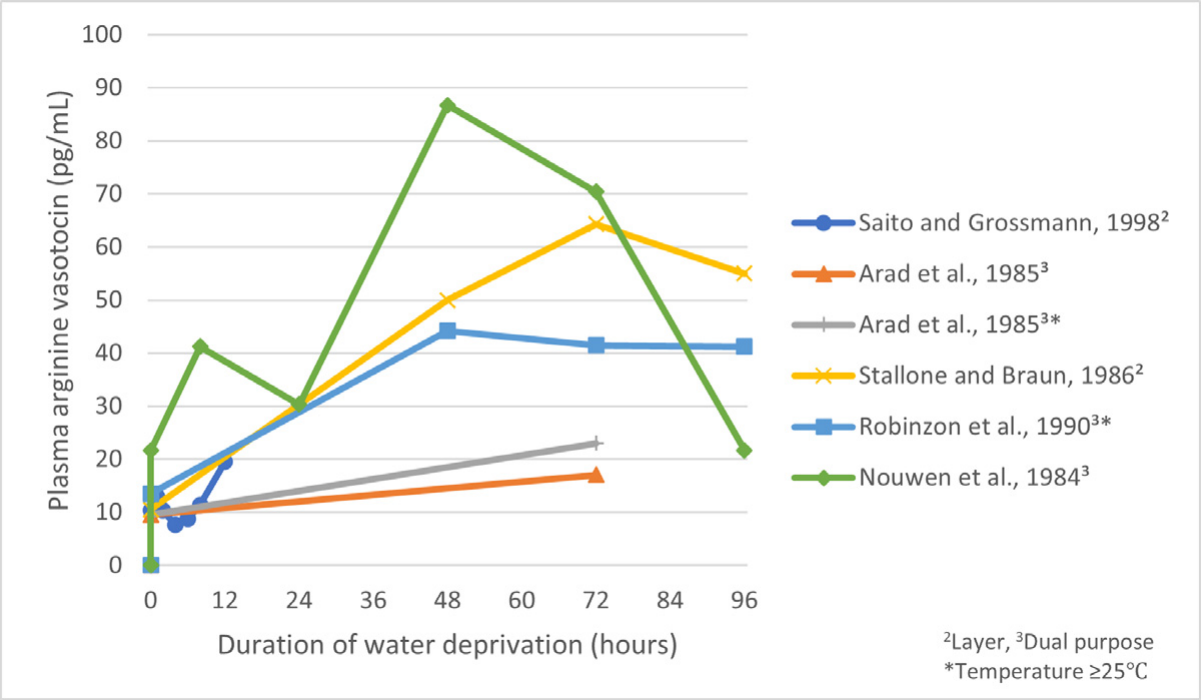
Plasma arginine vasotocin concentrations (pg/mL) reported by various studies across various durations of water deprivation. Points correspond with the time in which the measure was taken and are connected by a linear line for illustrative purposes, though in reality these relationships are not necessarily linear in nature. The shortest duration of deprivation measured was 0.5 h (Saito and Grossmann, 1998).

In response to increased plasma osmolality, AVT concentration becomes elevated in the plasma, and increased AVT mRNA concentrations become detectable in the hypothalamus (Koike et al., 1983; Saito and Grossmann, 1998). AVT concentration closely follows plasma osmolality concentration and may thus serve as another physiological indicator of dehydration. Saito and Grossmann (1998) found that 8 h of water deprivation (with measures taken at 0.5, 1, 2, 4, 6, and 8 h) was enough to significantly increase AVT in White Leghorn laying hens, while Nouwen et al. (1984) found that 24 h was needed in male Rhode Island Red chickens (Figure 2). An experiment by Robinzon et al. (1990) found that plasma AVT increased 3-fold during the first 24 h of water deprivation, but there was no further increase during the next 48 h in White Leghorn cockerels (housed between 25°C and 27°C) (Figure 2). Stallone and Braun (1986) observed a similar pattern in Rhode Island Red cockerels over a 96 h period (Figure 2). After 48 h of water deprivation of White Plymouth Rock chickens, significantly elevated AVT concentrations were observed (Arad et al., 1985) (Figure 2). The minimum duration of water deprivation to cause statistically significant changes in AVT concentrations from baseline was 8 h in laying hens (Saito and Grossmann, 1998).

**Figure 3:**
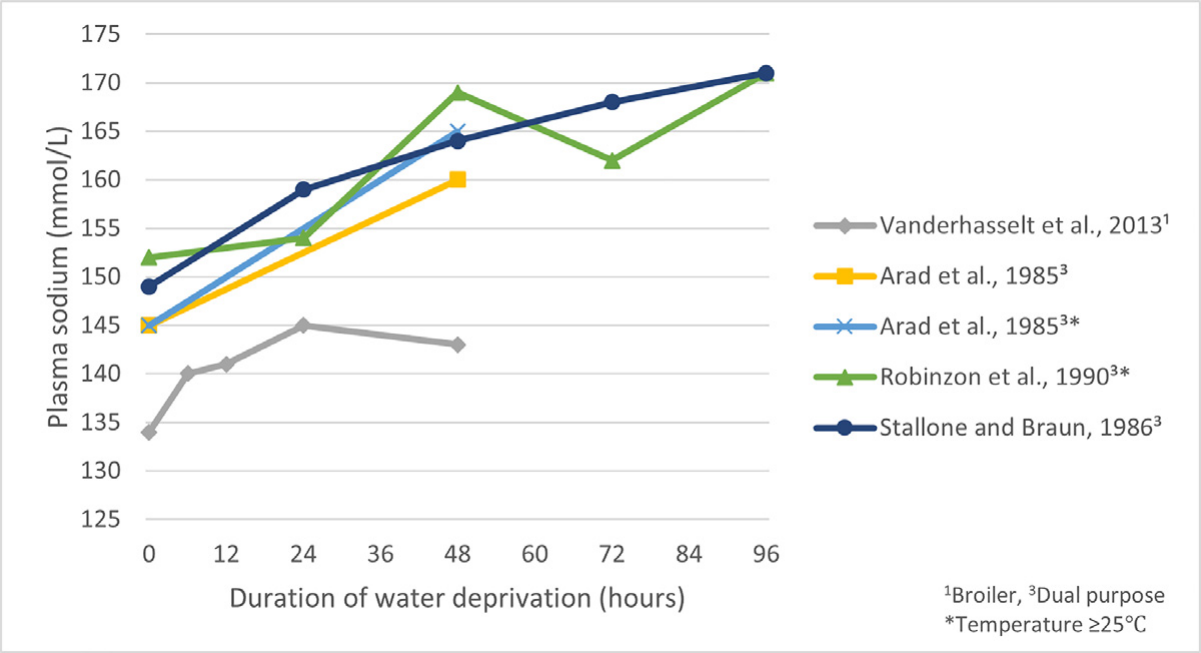
Plasma sodium concentrations (mmol/L) reported by various studies across various durations of water deprivation. Points correspond with the time in which the measure was taken and are connected by a linear line for illustrative purposes, though in reality these relationships are not necessarily linear in nature. The shortest duration of water deprivation assessed was 6 h (Vanderhasselt et al., 2013).

Poultry alter their balance of electrolytes, including sodium, chloride, and magnesium to maintain homeostasis during periods of dehydration (Frerichs et al., 2022). Whole blood sodium concentrations demonstrate the most consistent relation with duration of water deprivation, at least up until 24 h of deprivation (Vanderhasselt, 2013). Whole blood sodium concentrations in broilers steadily increased over a 24 h period of water deprivation (values measured at 0, 6, 12, and 24 h) and remained stable thereafter up to the final measurement at 48 h (Figure 3) (Vanderhasselt et al., 2013). Robinzon et al. (1990) found that plasma sodium concentration gradually increased over a 4-day period of water deprivation in White Leghorn cockerels (housed between 25°C and 27°C), with significantly elevated concentrations observed after 24 h, though it was the earliest time point measured (Figure 3). Arad et al. (1985) found significantly increased plasma sodium concentrations when White Plymouth Rock were subjected to a period of 48 h of water deprivation (Figure 3). In most studies, sodium concentration began to gradually increase immediately following removal of water, however statistically significant differences from baseline were not noted until after 24 h in broilers (Stallone and Braun, 1986; Vanderhasselt et al., 2013). Sodium concentrations are believed to be linked with the experience of thirst. In human studies, an increase of only 2 to 3% in plasma sodium concentrations has been shown to result in feelings of thirst (Stachenfeld, 2008). However, this relationship is not fully understood in poultry due to challenges associated with assessing thirst and a general lack of available studies on the topic.

Concentration of electrolytes, including plasma chloride, are expected to rise as a physiological response to dehydration that initiates a homeostatic response. Vanderhasselt et al. (2013) examined multiple potential indicators of thirst and dehydration in poultry and concluded that the plasma chloride concentration is a sensitive indicator of dehydration during the initial 6 h of water deprivation. Due to the tendency for chloride to level off after longer durations of deprivation, it is not recommended for use as an indicator of long-term dehydration. In one study, a significant increase of plasma chloride from approximately 295 to 345 mmol/L was observed in broiler chickens after 6 h of water deprivation, but this value did not significantly change after longer durations of deprivation (e.g., 12, 24, or 48 h) (Vanderhasselt et al., 2013). To our knowledge, no other studies have monitored the development of plasma chloride concentration as a response to water deprivation in poultry.

Magnesium is another electrolyte present in the blood and therefore would be expected to increase in response to water deprivation. Jakubowska et al. (2017) exposed birds to 3 pretransport fasting conditions for a 12-h period. The treatment group of birds deprived of both feed and water showed significantly lower blood magnesium concentrations than the treatment with only feed withdrawn and continued access to water. When comparing between a control group provided with both feed and water with the group deprived of both feed and water, no significant differences were observed, suggesting that 12 h of water deprivation did not impact serum magnesium concentrations (Jakubowska et al., 2017).

Renal filtration rate, in response to dehydration, may be indirectly measured through plasma creatinine concentration (Levy et al., 1990). Plasma creatinine may be a potential indicator which allows for discernment between dehydration, as a result of the transport process, vs. dehydration sustained prior on-farm, due to the length of restriction required to cause a significant rise in its concentration. In a study with broilers, creatinine concentrations were shown to decrease after 6 h of water deprivation from approximately 38 to 27 mmol/L, but to rise significantly over the next 42 h period at a linear rate, reaching 65 mmol/L after 48 h of deprivation (Vanderhasselt et al., 2013). In another experiment, laying hens were subjected to various levels of water restriction (0, 20, and 40% restriction) for a 1-wk period (Alamer and Ahmed, 2012). During this time, plasma creatinine concentration was elevated in those experiencing water restriction, suggesting reduced renal functioning (Alamer and Ahmed, 2012).

Birds conserve additional water during periods of dehydration through solute-linked water absorption in the lower intestine, in which prolactin likely serves as a regulatory hormone (Skadhauge, 2012). Arad et al. (1985) found that water deprivation of White Plymouth Rock chickens for a period of 48 h led to significantly increased plasma concentrations of prolactin, likely in response to increased plasma osmolality as well as sodium and chloride concentrations.

During dehydration, water exits the blood, leaving behind blood constituents, including protein molecules, thus leading to increased total plasma protein concentrations. In a study by Warriss et al. (1993), plasma total protein increased proportionally with plasma osmolality values over journey durations of 2, 4, and 6 h, suggesting its potential for use as an indicator for dehydration associated with transport durations of less than 12 h. However, these values did not reach levels significantly higher than baseline. Voslarova et al. (2011) examined pretransport handling and its effect on stress response in broilers. During 2 h of crating, the authors observed no significant changes in plasma total protein concentration.

**Figure 4:**
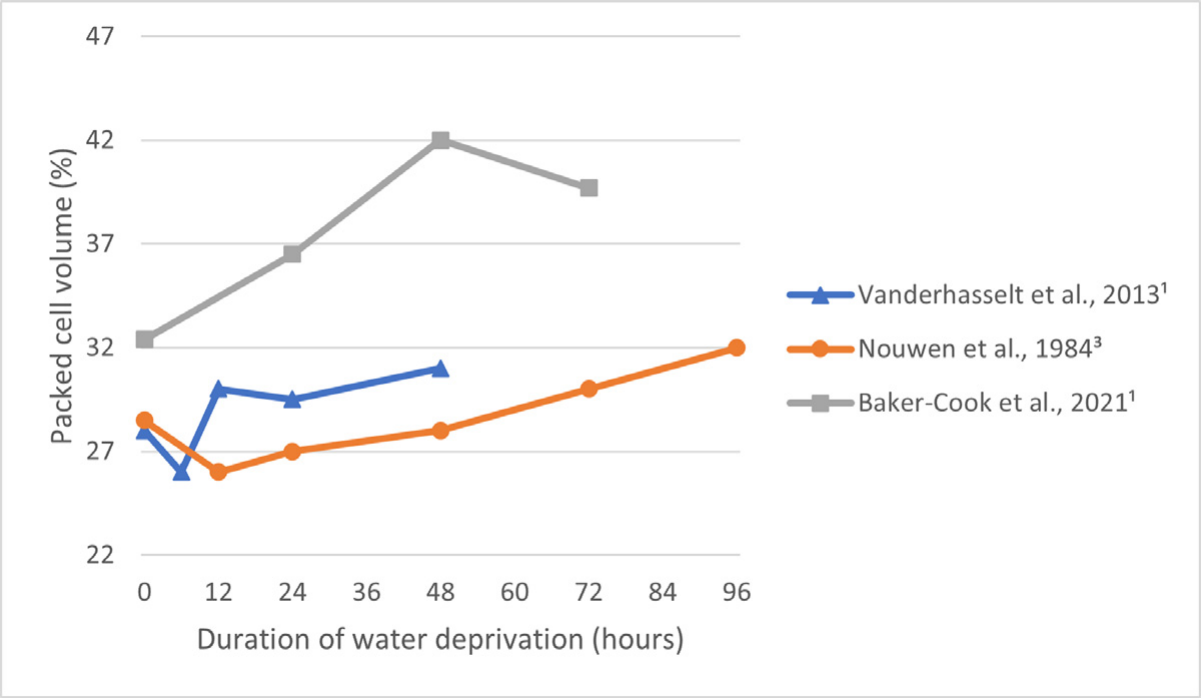
Packed cell volume (%) reported by various studies across various durations of water deprivation. Points correspond with the time in which the measure was taken and are connected by a linear line for illustrative purposes, though in reality these relationships are not necessarily linear in nature. The shortest duration of water deprivation assessed was 6 h (Vanderhasselt et al., 2013).

Packed cell volume measures the proportion of the blood that is made up of cells, of which most are red blood cells. Reduced blood plasma volume as a result of dehydration causes the proportion of red blood cells to plasma to rise (Zhou et al., 1999). Broilers at 50 d of age that were subjected to water-deprivation for 0, 24, 48, or 72 h had packed cell volume values that increased quadratically over time, reaching values significantly different from baseline at 48 h (Figure 4) (Baker-Cook et al., 2021). In another study with broilers, packed cell volume percentage initially dropped after 6 h of water deprivation before showing gradual increase up until 48 h of deprivation, though these values did not differ significantly from those of the control group (Figure 4) (Vanderhasselt et al., 2013). In a study with Rhode Island Red chickens, a slight drop in packed cell volume percentage was noted after 12 h, and then values gradually increased up until the study concluded at 96 h of water deprivation (Figure 4) (Nouwen et al., 1984). Iheukwumere and Hubert (2003) tested different water provision treatments of varying quantities in broilers and found that higher severity of water restriction led to higher packed cell volumes. When comparing across studies, the shortest duration of water deprivation that led to statistically significant increased packed cell volumes compared to baseline concentrations was found to be 48 h (Baker-Cook et al., 2021).

***Impacts on Indicators of Performance.*** Loss of body weight during transport, commonly referred to as “shrinkage,” is a well-documented phenomenon that results from water loss; with the principal driver of water loss being evaporative cooling (Warriss et al., 1990). This water loss not only impacts product weight but can contribute to poor meat quality (EFSA, 2011). Shrinkage has been shown to begin shortly after feed and water is withdrawn and body weight loss continues over time. For instance, Pan et al. (2018) found significant body weight losses of approximately 1.8% in broilers after 3 h of transport following an 8 h period of fasting overnight. Benibo and Farr (1985) found that dressed carcass weight of mixed sex broilers decreased (ls means decreased from 1,147–1,111 g) with increasing duration of withdrawal over 10 to 20 h of feed and water deprivation, with males showing greater body weight losses than females. Jacobs et al. (2017b) modeled weight loss during transport and reported body weight losses of 40.8 g/h. Multiple additional studies have demonstrated that broilers continue to lose body weight as the duration of feed and water deprivation increases. For instance, Wabeck (1972) found shrinkage percentages ranging from 1.29% after 4 h up to 4.93% after 24 h of deprivation. Veerkamp (1986) found shrinkage rates ranging from 0.2 to 0.25% per h, with younger birds being more negatively affected. Zhang et al. (2019) found live weight losses of 3.16% after 3 h vs. 1.79% after 0 h of transportation in a vehicle following a 9 h fasted period. For 36-day-old broilers, Baker-Cook et al. (2021) observed body weight losses of 236.6 g after 24 h and up to 379.5 g after 72 h of deprivation. Similar findings have been observed in turkeys with increasing body weight losses (range 13.55–42.65 g/kg of live weight) over periods of 4, 8, and 12 h (Duke et al., 1997).

Feed and water deprivation, coupled with stress from being transported can lead to reduced meat quality in poultry. Increased holding times at slaughter plants can further exacerbate these effects (Bianchi et al., 2006). Broilers that were transported on a vehicle for either 5-min or 4-h preslaughter had different meat quality traits. The birds exposed to longer journey times had reduced meat quality variables and signs of stress including lower initial pH (recorded 20 min postmortem), higher ultimate pH (recorded 24 h postmortem), higher postmortem temperature, and lower water holding capacity of their breast meat (Al-Abdullatif et al., 2021). Lower muscle pH can result from lactic acid build-up associated with transport stress and has been linked with lower water holding capacity and reduced tenderness of the product. When transported 100 km in length, corresponding to an approximate duration of 2.5 h, muscle concentrations of calcium, magnesium, sodium, potassium, phosphorus, and iron increased compared to broilers that were not transported but were still subjected to the same preslaughter handling (e.g., catching, loading, lairage), likely in response to losses of water from the body (Wójcik et al., 2009).

Substantial evidence suggests that longer journey and lairage durations lead to increased DOA rates; a common endpoint metric used to assess animal welfare during transport (Vecerek et al., 2006; Voslářová et al., 2007; Weeks et al., 2012; Saraiva et al., 2020). Gregory and Austin (1992) found that 5.7% of DOA broilers arriving at 6 processing plants in the UK were severely dehydrated. A study performing postmortem inspections at a slaughter plant in Portugal found that 2.68% of broiler carcasses showed signs of dehydration (“being dry, tacky and badly bled,” as described in Butterworth and Niebuhr, 2009; Saraiva et al., 2020). Based on transport of Dutch and German broiler chickens, it was reported that each additional 15 min of journey time resulted in an increase of 6% in DOA, and each additional 15 min spent in lairage resulted in a 3% increase in DOA (Nijdam et al., 2004). Based on data from Norwegian broiler flocks with either normal or high levels of DOA, it was found that the high-DOA group had average journey durations of approximately 1.5 h longer than the normal-DOA group (Kittelsen et al., 2017). Whiting et al. (2007) similarly found an association between journey time and DOA rate in Canada, with 10.5% of the DOA rate variability being attributed to the combination of the time of day in which slaughter was initiated, mortality rate on farm, and journey time. Another study from the Czech Republic found that journeys up to 50 km in length were associated with low DOA rates (0.15%), whereas this value increased to 0.86% for journeys 300 km or greater (Vecerek et al., 2006). While there does appear to be a clear link between journey length and DOA rates, it is important to be mindful of potential confounding effects with individual flocks or farms, as flocks from the same facility would not have been transported different distances from one another. It is also important to remember that water deprivation is only one of many stressors that poultry are subjected to during the transport process which may influence DOA rates.

***Contributing Factors.*** There are many factors that can exacerbate the effects of water deprivation and thus a bird's ability to cope with the stress of transport. For instance, the species and genetic line, environmental conditions during transport, and the bird's condition prior to transport can all impact an individual's response to water deprivation (Fisher et al., 2009). Below, selected factors are reviewed in relation to the development of thirst.

The environmental conditions during the different phases of transport can have a considerable impact on how quickly birds become dehydrated and may also impact the number of DOAs (Bayliss and Hinton, 1990; Nijdam et al., 2004). In broilers, water consumption has been shown to increase by about 7% for each 1°C above 21°C (NRC, 1994). When birds are dehydrated, their ability to cool themselves is greatly reduced, increasing their risk of hyperthermy and death (Mitchell and Kettlewell, 2004; Petracci et al., 2006; Whiting et al., 2007). Increased mortality has been observed during transport of broilers in summer months as compared to winter (Vecerek et al., 2006) as well as within hot and humid microenvironments on the transport vehicle (Mitchell and Kettlewell, 2009). So far, no studies have examined the effects of environmental conditions during transport on the interval from water deprivation to the initiation of thirst.

Poultry maintain their body temperature during heat stress through convection, conduction, radiation, and evaporative cooling, with the latter often resulting in bodily water loss which can lead to declines in plasma volume (Swayne and Radin, 1991; Kettlewell and Moran, 1992; Zhou et al., 1999). Further, changes in blood viscosity negatively impact tissue perfusion and circulatory distribution, thus reducing the ability of the birds to cool themselves through heat exchange surfaces (Zhou et al., 1998). In a study of commercial transport of broilers in Brazil, the application of a water mist treatment prior to transportation was shown to reduce DOA rates from between 0.16 and 0.27% to 0.12 and 0.17% (Silva et al., 2011). In an EFSA (2011) scientific opinion, it was suggested, based on expert opinion, that risk of death under conditions of thermal stress is greater in journeys over 4 h for both broilers and end-of-lay hens than in journeys lasting less than 4 h. Therefore, if journeys are over 4 h, it was recommended to use transport vehicles equipped with mechanical ventilation systems to mitigate the impacts of hot and humid environments (EFSA, 2011; Weeks et al., 2019). However, this may be challenging to implement as most vehicles currently used in the EU for the transport of poultry are passively ventilated, as described in Mitchell and Kettlewell (2008).

Birds that have difficulties reaching water while on farm are at an increased risk of experiencing dehydration during transport due to them not being fully hydrated at initiation of catching. Gregory and Austin (1992) reported that of severely dehydrated DOA broilers, 24% of them weighed less than 1 kg. The authors speculated this was due to runts being unable to reach drinkers as the water lines are raised over the lifetime of the flock to account for growth. Walking impairments, which are highly prevalent in fast-growing meat poultry strains (Hocking, 2014; Hartcher and Lum, 2020), can also contribute to birds struggling to reach water lines. Birds with severe gait abnormalities that impair their mobility have been found to have higher plasma osmolality, indicating dehydration (Butterworth et al., 2002). Lund et al. (2013) found that roughly half of the broilers that arrived at the slaughter plant with kidney disease and signs of dehydration also demonstrated compromised mobility when inspected at slaughter, suggesting that gait impairments may contribute to chronic dehydration on farm.

In addition, other factors such as illness, injury, or social stress can reduce or even inhibit access to water, thus placing birds at a heightened risk of dehydration associated with transport (Butterworth and Weeks, 2010; Savory, 2010). Availability of water may also impact laying hen hydration prior to transport, with decreased water consumption being linked with increased stocking densities and decreased water nipple availability (Feddes et al., 2002).

In recent years, the modern fast-growing strains of broilers have been characterized and shown to differ from slower growing broilers in many aspects. In addition to differences in musculoskeletal structure, modern, heavily selected strains may also have increased needs for water to keep up with their fast metabolism, as water requirements are closely linked with feed consumption (Viola et al., 2009; Ahmed and Alamer, 2011). Similarly, high-yielding laying hens may also be more sensitive to water deprivation than lower-yielding hens due to their increased water needs to produce eggs (Saito and Grossmann, 1998). Poultry that have adapted to very hot and dry regions have been shown to have higher tolerances to water deprivation than commercial strains (Ahmed and Alamer, 2011; Chikumba et al., 2013). In Ahmed and Alamer (2011), a local Saudi breed of laying hen and a commercial strain were subjected to 0, 20, or 40% water deprivation over a 3-wk period. Body weight and egg production decreased in both strains, but declines were delayed by 1 wk in the local breed. Authors hypothesized that this could be a reflection of the lower water requirements of the local breed due to their lower metabolic rate and also potentially be linked to improved capability to budget body water resources. Chikumba et al. (2013) restricted water to 2 strains of hens prevalent in Southern Africa (Naked Neck and Ovambo) and found that both strains could tolerate water restriction up to 40% over a 60-day period as assessed by hematological and biochemical responses, however the Naked Neck birds seemed to tolerate the stress better, likely due to their increased heat tolerance as a result of reduced plumage cover.

## DISCUSSION

To ensure good welfare during transport of poultry to slaughter, the total time that birds go without access to water needs to be considered, beginning when water is first shut off in the barn, continuing through catching, loading, the journey on the vehicle, lairage, unloading, and up until the birds are killed. As is evident from the review of the literature, studies regarding the effects of water deprivation on poultry welfare during transport are scarce, and the vast majority of studies focusing on consequences of water deprivation have been conducted with broiler chickens housed on farm or under experimental conditions. Additionally, in many of the studies the birds were provided ad libitum access to feed during the water deprivation period. This could have influenced the onset or severity of the physiological and behavioral responses and would not be representative of conditions experienced under commercial transport. There is, thus, insufficient data to determine the exact duration of time that poultry may go without water in connection with transport before their welfare begins to deteriorate.

The point in which birds begin to experience negative emotional states in relation to the extent of water deprivation is unclear, though the few existing motivation testing and behavioral studies may offer some suggestions. After as little as 2 h of water deprivation, increased levels of redirected aggression within a test arena have been noticed in laying hens (Haskell et al., 2004), which may suggest that birds begin to experience negative emotional states around this time. Compensatory water consumption in broilers was observed on farm after 6 h of water deprivation. However, this was the shortest duration studied and thus it cannot be concluded that birds are not motivated to drink prior to this time (Sprenger et al., 2009). Similarly, in motivation tests, the shortest duration of water deprivation studied in the home pen was 12 h (Rault et al., 2016), making conclusions on the onset of thirst based on available behavioral tests currently impossible. More research is needed to exploit the great potential for determining the effect of water deprivation on bird welfare by using behavioral motivation tests, especially under transport conditions, where factors such as duration of water deprivation (using periods corresponding to typical transport durations, i.e., under 12 h), environmental conditions (particularly temperature and humidity), stocking density, and animal-based factors such as walking ability are considered. A recent study employing conditioned place aversion in dairy calves provides an example of how motivational testing may be used to assess affective states associated with transport conditions in livestock (Creutzinger et al., 2022).

Physiological indicators of severe dehydration are available. By the time birds are in a severely dehydrated state their welfare is most likely also severely compromised. Thirst, defined as a negative motivational state, likely begins when the bird is motivated to drink but fails to access water, and increases in strength becoming a negative emotional state as the threat to fitness (e.g., risk of severe dehydration) increases (Fraser and Duncan, 1998). Determining the point at which poultry welfare begins to deteriorate due to water deprivation is difficult, and further research is needed, as well as the development of ABMs that are feasible for use during assessment at transport.

When examining available physiological indicators of dehydration, it has been suggested by Vanderhasselt et al. (2013) that measures of plasma osmolality, plasma AVT concentration, and plasma chloride concentration are the most suitable variables for detecting significant differences in treatment groups compared to control groups (i.e., 0 h water deprivation) due to their observable fluctuations occurring within the first 12 h; a duration that corresponds with typical transport times. Based on the current available literature, the earliest timepoint in which broilers’ physiology changed to a significant extent to preserve water was 6 h when using plasma chloride as an indicator (Vanderhasselt et al., 2013). It is possible that this rise in chloride concentration is linked with thirst, though in this same study the plasma sodium concentration did not show significant increases until 24 h of water deprivation. Further, in this study the birds were not subjected to fasting as they would be under transport conditions, thus making interpretation difficult. The above conclusion is in line with the EFSA AHAW Panel (2022) based on available literature and expert opinion in which they concluded that when poultry are kept in their thermoneutral zone, water deprivation of periods longer than 6 h (based on plasma chloride concentration), and for periods over 12 h (based on creatine response) will lead to thirst, with greater certainty in their conclusion of 12 h vs. 6 h. The increased water necessary to produce eggs as compared to growth, coupled with typically longer journey times required to reach suitable slaughter plants, means that end-of-lay hens may be more likely to be negatively impacted by water deprivation upon arrival than broilers (EFSA AHAW Panel, 2022).

It is important to stress that the timing of these physiological responses relative to thirst is unknown. Due to this uncertainty, a risk reductive approach would be to restrict the total time that birds are deprived of water to 6 h, though there is still the possibility that thirst occurs even earlier. On the other hand, some studies did not observe significant physiological responses until after 24 h or longer, suggesting that birds may be able to cope with longer periods of water deprivation under certain conditions. To make informed inferences on the relationship between thirst and transport durations, more information is needed regarding the impacts of water deprivation of shorter durations, such as less than 6 h, which is currently lacking, and as the understanding of the interaction between the state of dehydration, the feeling of thirst, and the experience of negative emotional states improves. When determining how long poultry can go without access to water before their welfare begins to deteriorate, it is critical to also consider the thermal environmental conditions (Nawab et al., 2018; El Sabry et al., 2023). The vast majority of the available studies, and the data that were utilized in this review, were conducted under thermoneutral conditions. It is clear that thermal stress increases the negative impacts of water deprivation by increasing water losses through evaporative cooling (Sayed and Downing, 2011), calling for research that includes considerations as to how thermal conditions as well as conditions typical of transport influence the maximum duration of water deprivation before poultry welfare is negatively affected. In light of the ongoing climatic changes, focus on the consequences of more extreme climatic conditions are needed (El Sabry et al., 2023).
